# Perforated Stercoral Ulceration of Transverse Colon in the Setting of Multiple Comorbidities: A Case Report

**DOI:** 10.7759/cureus.79359

**Published:** 2025-02-20

**Authors:** Peter Richa, Adam K Bobak, Carter R Olberding, Joseph R Hartigan

**Affiliations:** 1 School of Medicine, Lake Erie College of Osteopathic Medicine, Jacksonville, USA; 2 School of Medicine, Lake Erie College of Osteopathic Medicine, Bradenton, USA; 3 General Surgery, Ascension Health/St. Vincents, Jacksonville, USA

**Keywords:** diverticulosis, intestinal perforation, small bowel diverticulitis, small bowel diverticulosis, stercoral colitis, stercoral perforation, stercoral ulcer, stercoral ulcer perforation

## Abstract

Stercoral colitis is an inflammatory condition resulting from the long-term collection of fecal matter that may lead to colonic ulceration and perforation, consequently representing a surgical emergency. Herein, we present the case of a 65-year-old female who developed stercoral ulceration of the transverse colon, an uncommon location of occurrence. The patient had multiple comorbid conditions and risk factors for stercoral colitis that included a history of constipation, major depressive disorder, hypothyroidism, and a partial colonic resection due to an upper rectal perforation secondary to diverticulitis. With features of peritonitis on physical examination, historical, and computed tomography findings, the patient underwent urgent open abdominal exploration with colonic resection of the impacted bowel and end transverse colostomy. The patient had a complicated postoperative course owing to a respiratory infection and intraabdominal abscess formation but adequately recovered to be discharged home. Thus, urgent surgical intervention is necessary for survival in patients with stercoral ulceration, but modification of risk factors may be crucial in preventing stercoral colitis and its potentially fatal complications.

## Introduction

Stercoral colitis is a rare, acute inflammatory condition that results from chronic fecal impaction and derives its name from the Latin term for feces: *stercus *[1]. Stercoral colitis is characterized by the collection of impacted colonic fecal material (fecaloma) with subsequent colonic distention and inflammation [2]. Without adequate treatment, the increased colonic pressure and distention can compromise vascular supply, causing ischemic necrosis and, if severe, perforation with fecal contamination throughout the peritoneal cavity.

Stercoral colitis can present in various sites throughout the colon, but ulceration most commonly occurs in the rectum and sigmoid colon [2]. These areas are more susceptible to ulcers due to minimal collateral circulation, a relatively narrower lumen, and decreased water content of stool within these colonic segments [3]. While stercoral colitis can be found throughout the entire population, the mean age of onset is individuals over 60, especially those with comorbidities predisposing them to constipation [4]. These comorbidities may include patients with chronic opioid use, are in a bedridden state, or have psychiatric conditions [1]. The clinical presentation of stercoral colitis can significantly vary among patients. Classically, patients may present with abdominal tenderness, distention, nausea, and vomiting with a lack of diarrhea. However, in the setting of complicated stercoral colitis, clinical presentation may indicate sepsis with febrile, hypotensive patients with elevated white blood cell counts [1,5]. Due to the variability in presentation, imaging and lab values are important diagnostics tools. Furthermore, a comprehensive medical history review may be essential in providing information about the patient’s presenting condition. An abdominal computed tomography (CT) is the most sensitive and specific study, displaying stool in the rectal vault and possible perforation [2]. The CT scan can help distinguish stercoral colitis from similar pathologies such as diverticulitis and classic bowel obstruction [1]. As stercoral colitis and diverticular disease may coexist in a patient, the clinical picture may become complicated in distinguishing the two pathologies [3]. Nonetheless, specific features such as peri colonic fat stranding, diffuse bowel wall edema, and the presence of fecalomas can aid in diagnosing the condition at hand [1].

Management of stercoral colitis is determined by the presence or absence of colonic perforation. In the setting of perforation, open laparotomy, peritoneal cavity lavage, and Hartmann’s procedure are the necessary treatment steps to generate the best outcome [3]. Patients should be resuscitated with IV fluids as needed and given broad-spectrum IV antibiotics [1]. On the other hand, medical management of uncomplicated stercoral colitis does not require operative therapy. Conservative treatment measures include manual disimpaction of the fecal mass, an oral bowel regimen, and close monitoring [1]. Notably, patients should remain nil per os (NPO), and opioids should be avoided [2]. Here, we report a case of a 65-year-old woman with a previous rectal perforation secondary to diverticulitis, now diagnosed with complicated stercoral colitis of the transverse colon that required immediate surgical intervention.

## Case presentation

Our patient is a 65-year-old female who presented to the emergency department with acute onset, sharp abdominal pain, and one episode of non-biliary emesis. Notably, the patient presented to the emergency department with similar symptoms 7 years prior. At that time, exploratory laparotomy revealed that the patient had experienced an upper rectal perforation secondary to diverticulitis, and a low anterior resection was conducted with colostomy bag creation. Four months after her initial low anterior resection, the patient underwent a screening colonoscopy that revealed diverticulosis of the descending, transverse, and ascending colon. In addition to her low anterior resection and diffuse diverticulosis, past medical and surgical history included colitis, multiple unspecified colon surgeries due to diverticulitis, chronic obstructive pulmonary disease (COPD), atrial fibrillation, cardiomyopathy with reduced ejection fraction, generalized anxiety disorder, major depressive disorder, dyslipidemia, hypothyroidism, gastroesophageal reflux disease (GERD), anemia, and alcohol and tobacco abuse. The patient reports a long history of constipation, but opioid use was definite.

**Figure 1:**
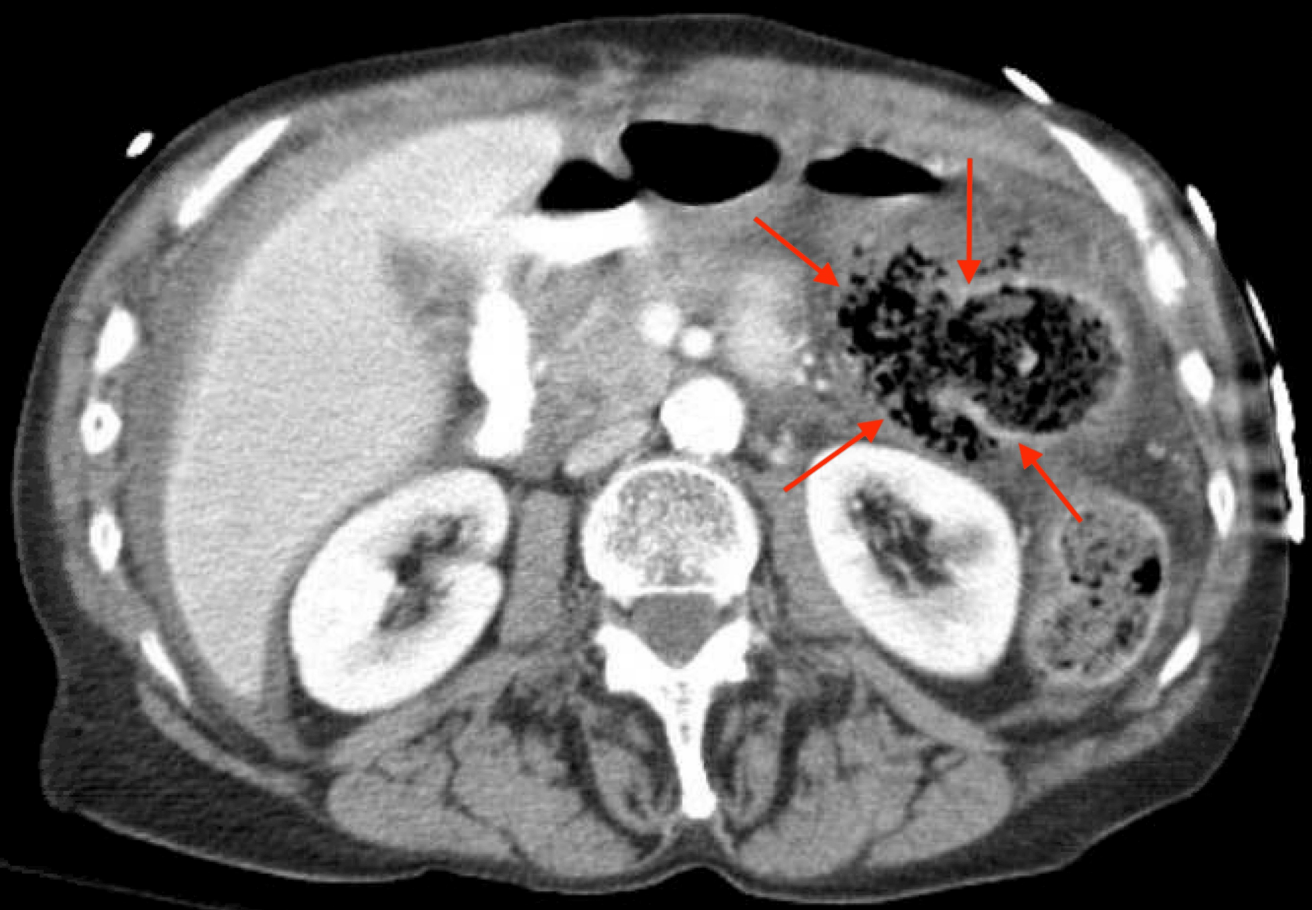
Axial computed tomography of abdomen and pelvis with contrast, showing a perforated viscus in the distal colon (red arrows) with intraperitoneal free air

**Figure 2:**
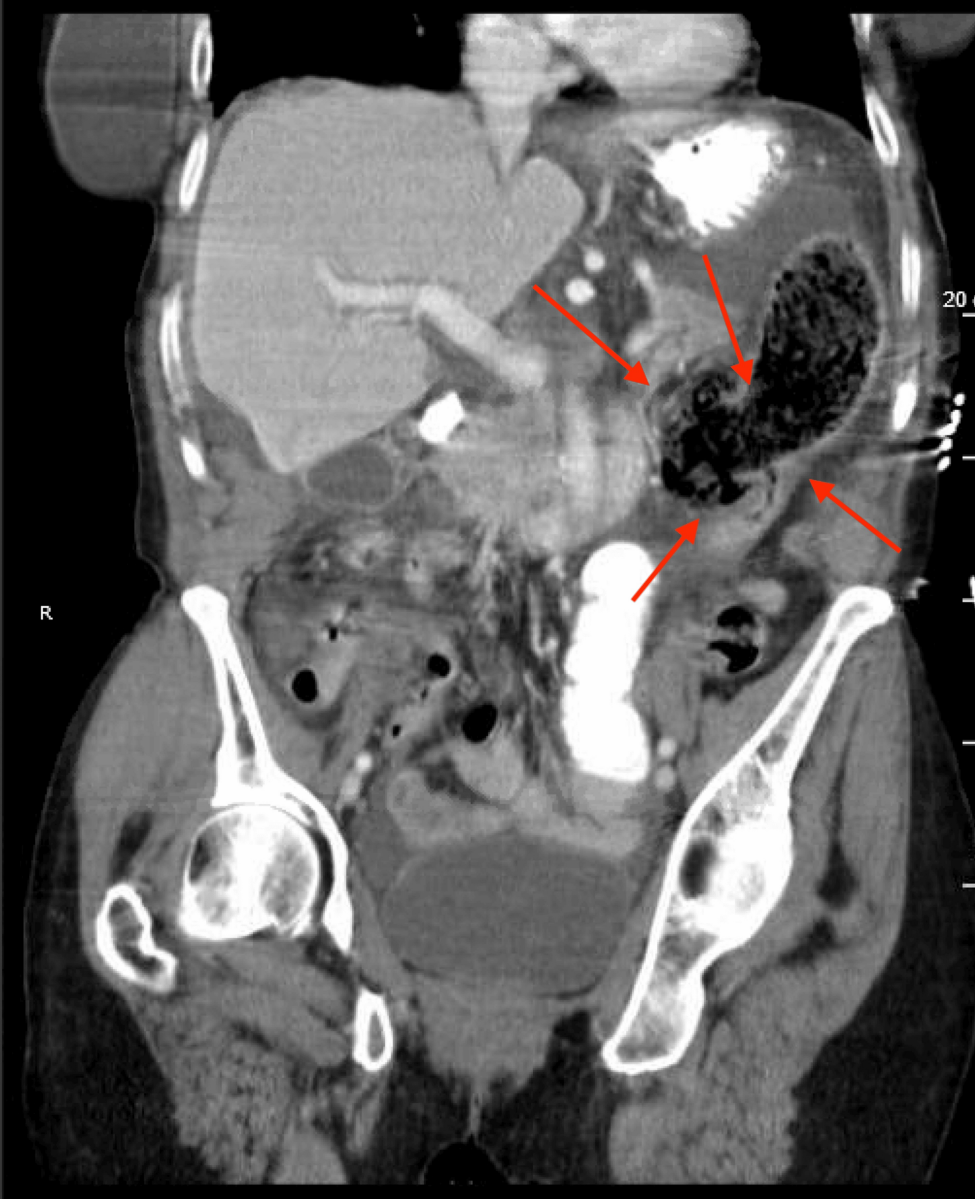
Coronal abdomen and pelvis with contrast, showing a perforated viscus in the distal colon (red arrows) with intraperitoneal free air

At the present visit, a physical exam revealed a soft, distended abdomen with absent bowel sounds. An abdominal CT scan revealed intraperitoneal free air consistent with a perforated viscus, moderate volume ascites, and mesenteric edema (Figures 1-2). The distal transverse colon or splenic flexure was proposed to be the perforation site due to the presence of a large air-containing structure protruding at the colonic wall at this site. At that point, immediate surgical intervention was indicated as the patient showed signs of sepsis with a blood pressure of 70/50 mmHg, a respiratory rate of 30 breaths/min, and CT evidence of bowel perforation.

An open abdominal exploration was performed with a midline incision to enter the abdominal cavity. The abdomen was copiously irrigated with saline, and the transverse colon was mobilized from the omentum. A large portion of the colonic wall was infarcted near a perforation site of the distal transverse colon, and free stool was observed throughout the abdominal cavity. Dissection of the colon was continued, and hard stool could be noted throughout and at the perforation site, indicating the presence of a stercoral ulcer. The colon was further dissected proximally in the direction of the liver and distally toward the sigmoid colon to identify regions of infarction. The infarcted segment of the colon was removed using the gastrointestinal anastomosis (GIA) green load and EnSeal device, and the final specimen was 28 cm in length by 4 cm in diameter. Two French drains were placed; one was located above the spleen, and the other could be found extending into the pelvis. Lastly, a colostomy was performed in the right abdomen, followed by wound closure. The open abdominal exploration confirmed the presence of a large perforation within the distal transverse colon secondary to stercoral ulceration. The patient was then admitted to the hospital for postoperative observation and recovery.

**Figure 3:**
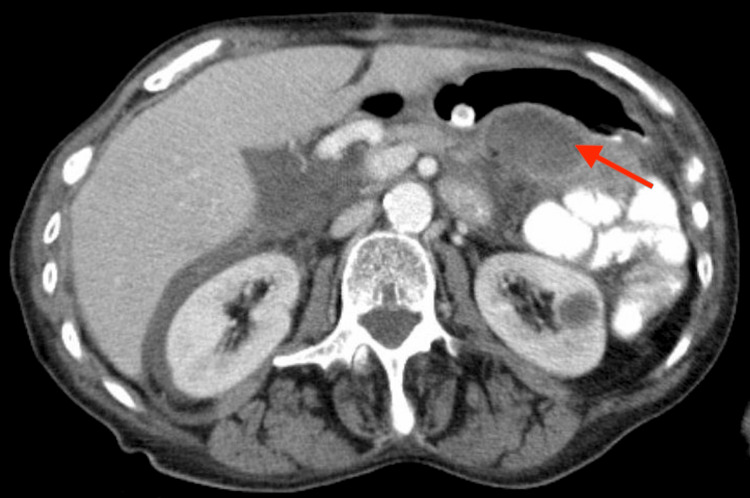
Axial computed tomography view of the abdomen and pelvis with contrast, showing the postoperative peritoneal abscess (red arrow)

Numerous complications were encountered during her postoperative hospital stay and included respiratory failure secondary to pneumonia, intraabdominal abscesses, atrial fibrillation with rapid ventricular response, acute metabolic encephalopathy, and upper extremity deep vein thrombosis. Approximately 26 days after the initial presentation and surgical procedure, the patient was stable to be discharged to a short-term rehab facility. However, the patient returned to the hospital 15 days after discharge due to pain surrounding the colostomy site. The patient was found to be afebrile with low blood pressure and elevated white blood cell count. An abdominal CT displayed the previous intraabdominal abscess along with fat stranding, intraabdominal fluid, and possibly worsening peritonitis (Figure 3). At that time, there were no indications for immediate surgical intervention, and the patient was managed with analgesics, bowel rest, antibiotics, and fluids.

## Discussion

Stercoral colitis may have vague presenting symptoms that include abdominal pain, nausea, and vomiting. Additionally, presentation is variable as one study reports that as many as 60% of patients with stercoral colitis who present to the emergency department do not have abdominal pain [6]. Thus, stercoral colitis is a challenging condition to diagnose and has a wide list of differentials. The differential diagnosis of stercoral colitis includes diverticulosis, large bowel obstruction, ulcerative colitis, infectious colitis, malignancy, acute mesenteric ischemia, intraabdominal abscess, and others [5]. Similarly challenging, patients may have a past medical history corresponding to one or many of these differential diagnoses as occurred in our patient who has had diverticulitis.

Diverticulitis and stercoral colitis share many risk factors that include low fiber intake and opioid use, but management of the two conditions differs [7,8]. Therefore, it is pertinent to utilize the most sensitive and specific modality, an abdominal CT scan, to confirm the diagnosis [2]. In stercoral colitis, the abdominal CT will display colonic distention, colonic wall thickening, diffuse wall edema, and possible pressure necrosis or ulceration [5]. While an abdominal CT is also the modality of choice to diagnose diverticulitis, the most common findings are wall thickening, pericolic fat stranding, and, most importantly, inflamed diverticulum [9]. Considering the similar presentations and findings of stercoral colitis and diverticulitis but differences in management, it is necessary to differentiate them. This is especially true in cases of comorbid stercoral colitis and a prior history of diverticulitis, as seen in our patient. Properly differentiating stercoral colitis from the vast array of other conditions on the differential diagnosis can be crucial in initiating treatment and improving survival.

In one retrospective study of patients across the United States who had CT evidence of stercoral colitis, a large percentage of patients (31.2%) were discharged home without admission to the hospital [6]. More than half of these patients had related complications within three months that included necessary surgical intervention, return to the emergency department, and death [6]. Similarly, Bae et al. (2024) state that mortality rates due to stercoral colitis may exceed 63% in the setting of severe complications, such as sepsis [1]. Another retrospective case study mirrors these findings by stating that in patients with stercoral colitis and/or fecal impaction, 90% with diagnoses of sepsis had expired in the hospital [10]. Of the patients in the study who had perforated stercoral ulcers, the majority had passed away or been moved to hospice care [10]. Thus, the potential consequences of stercoral colitis are grave, and risk factors for development that include constipation, psychiatric conditions, and opioid abuse should be mitigated.

Chronic constipation is the largest risk factor for the development of stercoral colitis and has a high prevalence in the elderly population, who are most at risk for complicated stercoral colitis [1,8]. Thus, conditions that can lead to constipation may eventually progress to stercoral colitis. One of the most common gastrointestinal complications of hypothyroidism is constipation due to reduced gut motility, which can lead to the overgrowth of colonic bacteria [11]. Likewise, psychiatric conditions, such as schizophrenia and major depressive disorder, are associated with constipation, with prevalence rates up to 36.3% and 57.7%, respectively [12]. Drugs commonly used to treat psychiatric conditions, including clozapine and tricyclic antidepressants, also predispose patients to developing constipation [8,13]. On the other hand, the potential role of cigarette smoking in the formation of stercoral ulcers is more complex.

Cigarette smoking is known to have a causative role in the formation of gastric ulcers as it can increase endothelial dysfunction in microvasculature, resulting in ischemia and fibrosis [14]. Similarly, smoking can alter inflammation and the immune response of the bowel’s lining [14]. Though its effects are known in gastric ulcer formation, less is known about its risk in stercoral ulceration. Some studies have reported no effect of nicotine on rectal muscle tone, but constipation is a frequently reported symptom of nicotine withdrawal [15,16]. Thus, nicotine may play a protective role against stercoral colitis as it does in ulcerative colitis, and nicotine withdrawal may be a risk factor for stercoral colitis [14]. Though nicotine’s role in gastrointestinal motility requires more study and is not an established risk factor for stercoral colitis, decreased bowel motility is a risk for constipation and stercoral ulceration.

Surgical interventions may alter intestinal structure and bowel motility [17]. With the possible fibrosis that ensues, stercoral ulcer perforation may be forthcoming in a patient’s future if colonic motility is impacted [17]. The surgical intervention needed to treat our patients’ previous perforated diverticulitis may have initiated the development of risk factors for stercoral ulcer development. Together, all of these risk factors may increase the risk of serious stercoral colitis with complications like ulceration and sepsis, as occurred in our patient.

The patient reported in this case report had multiple of the risk factors mentioned for the development of stercoral colitis, including but not limited to constipation, major depressive disorder, hypothyroidism, and prior surgical intervention. These factors may have contributed to the patient’s presentation with stercoral ulceration in an uncommon location: the transverse colon near the splenic flexure [18]. Though this is the case, future studies are necessary to understand if these multiple comorbid factors contributed to the formation of the ulcer in the transverse colon. Likewise, the role of smoking in stercoral ulceration warrants further study. This case demonstrates such a rare yet possible set of circumstances, requiring immediate treatment and monitoring. This case also highlights the importance of proper diagnosis and swift treatment of stercoral ulceration, an uncommon but highly morbid condition.

## Conclusions

Stercoral colitis with ulceration was diagnosed through physical exam, CT, historical findings, and ultimate exploratory laparotomy in the presence of various risk factors. The differentiation of stercoral colitis from similar conditions through CT findings may prevent progression to stercoral ulceration as occurred in this patient. Swift diagnosis and treatment with open abdominal exploration and removal of ulcerated tissue and fecalomas were necessary for the survival of this patient. This case report highlights the urgent surgical nature of stercoral ulceration and its various risk factors. This case also raises the question of additive effects of risk factors on the development of stercoral ulceration in uncommon locations, like the transverse colon. Though stercoral colitis can be a challenging diagnosis with numerous differentials and vague presenting symptoms, rapid diagnosis is necessary to modify risk factors before life-threatening complications like ulceration experienced by this patient can occur.
